# Supplementary figures and images for: A mutagenesis screen for essential plastid biogenesis genes in human malaria parasites
Source: PLoS Biol. 2019 Feb 6;17(2):e3000136. doi: 10.1371/journal.pbio.3000136 (PMC6380595; doi:10.1371/journal.pbio.3000136)

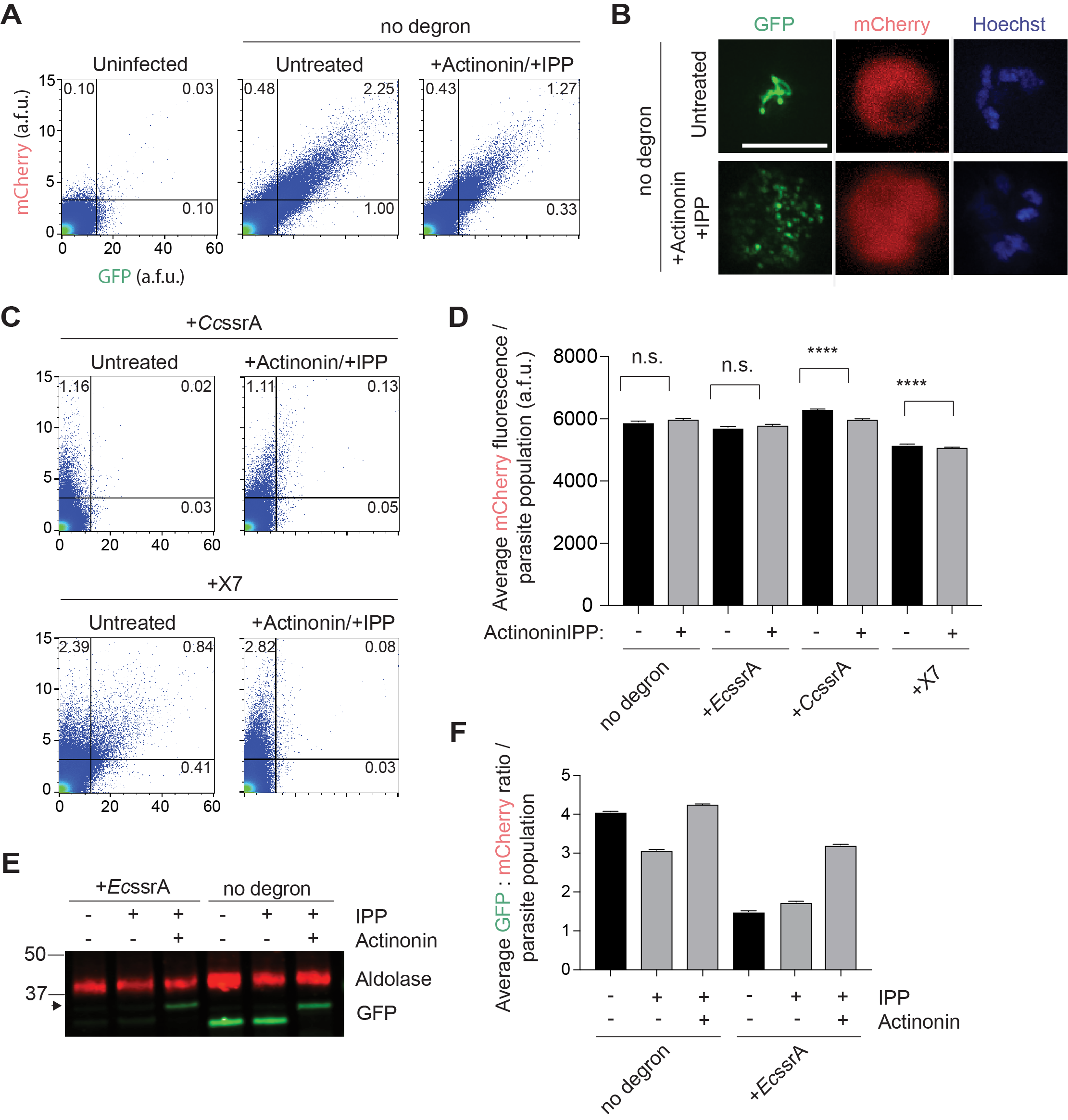

Supplement: S1 Fig — (A) Flow cytometry plots showing mCherry and GFP fluorescence in untreated versus actinonin/IPP-treated parasites expressing ACPL-GFP with no degron tag. The percentage mCherry+, GFP+ parasites in the population is indicated. Uninfected RBCs were used to set gates for mCherry and GFP fluorescence. (B) Representative live-cell fluorescent images of untreated and actinonin/IPP-treated parasites expressing mCherry and ACPL-GFP. Hoechst stains for parasite nuclei. Scale bar 5 μm. (C) Flow cytometry plots showing mCherry and GFP fluorescence in untreated versus actinonin/IPP-treated parasites expressing ACPL-GFP-CcssrA or ACPL-GFP-X7. The percentage mCherry+, GFP+ parasites in the population is indicated. Uninfected RBCs were used to set gates. (D) Average mCherry fluorescence of reporter strain populations. Data are shown as mean ± SEM (n = 3). ****P < 0.0001, unpaired two-tailed t test. Tabulated data are shown in S1 Data. (E) GFP protein levels in untreated versus IPP only, or actinonin/IPP-treated parasites expressing GFP-EcssrA (n = 1 experiment). (F) Ratio of GFP: mCherry fluorescence in untreated versus IPP only, or actinonin/IPP-treated parasites expressing ACPL-GFP-degron. Data are shown as mean ± SEM (n = 1 experiment). Tabulated data are shown in S1 Data. (TIF) [file pbio.3000136.s001.tif]

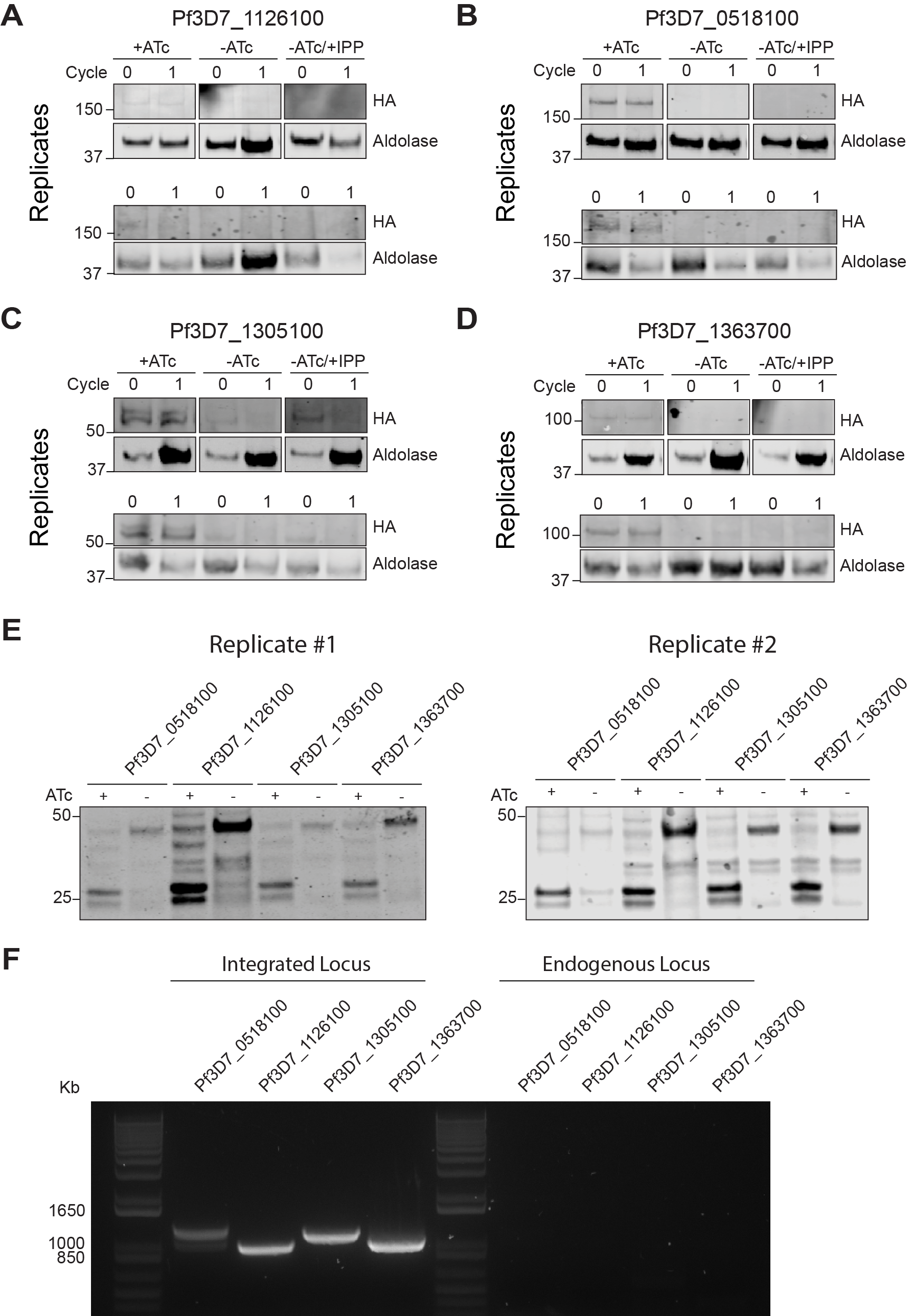

Supplement: S2 Fig — Individual replicates of western blot of HA-tagged protein candidates in TetR/DOZI parasite strains in +ATc, −ATc and −ATc/+IPP parasites. Protein levels for the initial and first reinvasion cycles are shown (0 and 1, respectively). Aldolase serves as a loading control. (A) Pf3D7_1126100 (Atg7), (B) Pf3D7_0518100 (conserved unknown), (C) Pf3D7_1305100 (conserved unknown), and (D) Pf3D7_1363700 (conserved unknown). (E) Individual replicates of full western blots showing ClpP processing for all candidates. (F) PCR analysis of genomic integration of TetR/DOZI plasmid in parasite strains for each individual candidate. (TIF) [file pbio.3000136.s002.tif]

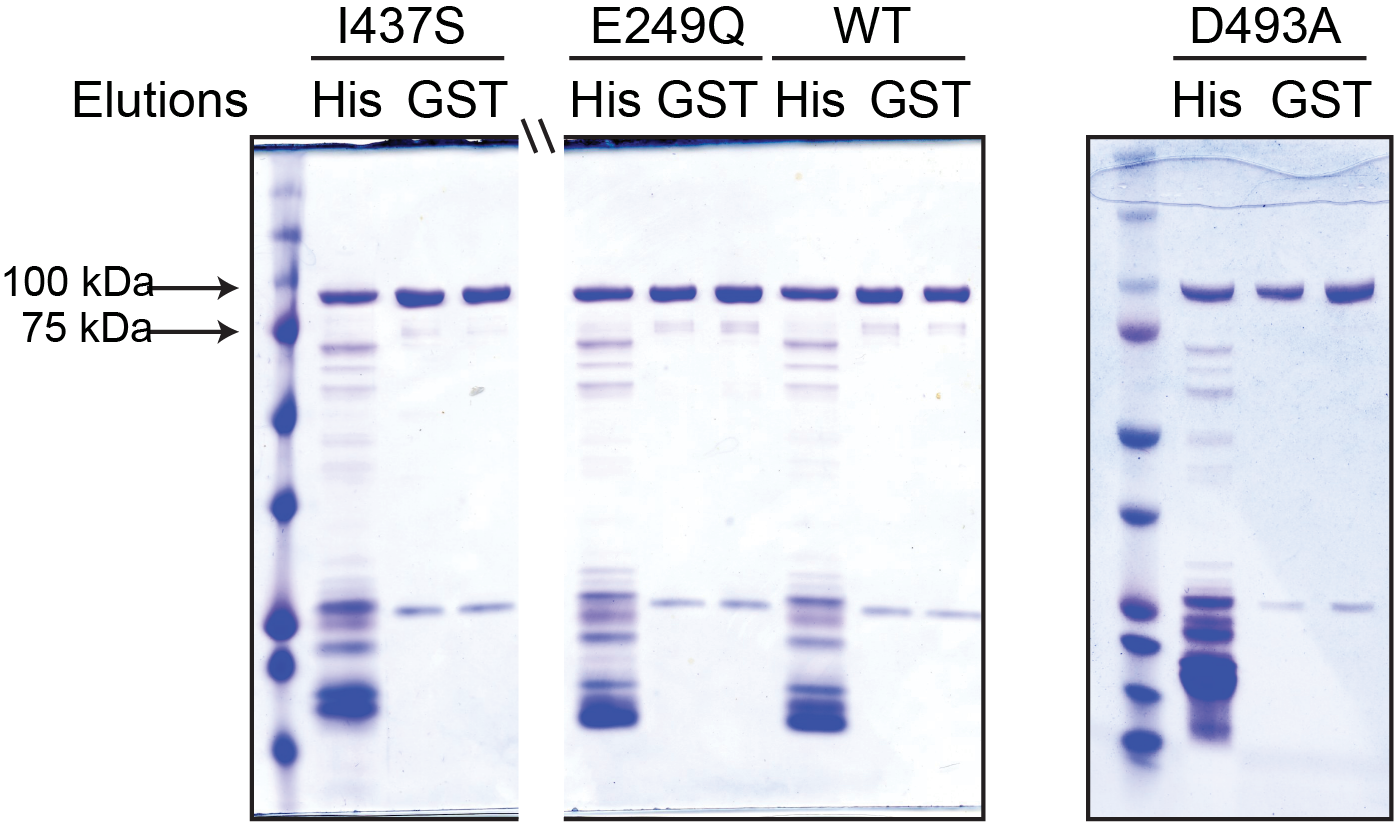

Supplement: S3 Fig — His6-SUMO-PfFtsH191-612-GST WT and corresponding variants were expressed from a pET22-based vector. A two-step affinity purification was performed for each recombinant enzyme (incubation and elution from Ni-NTA resin, followed by incubation and elution from GSTrap column). (TIF) [file pbio.3000136.s003.tif]

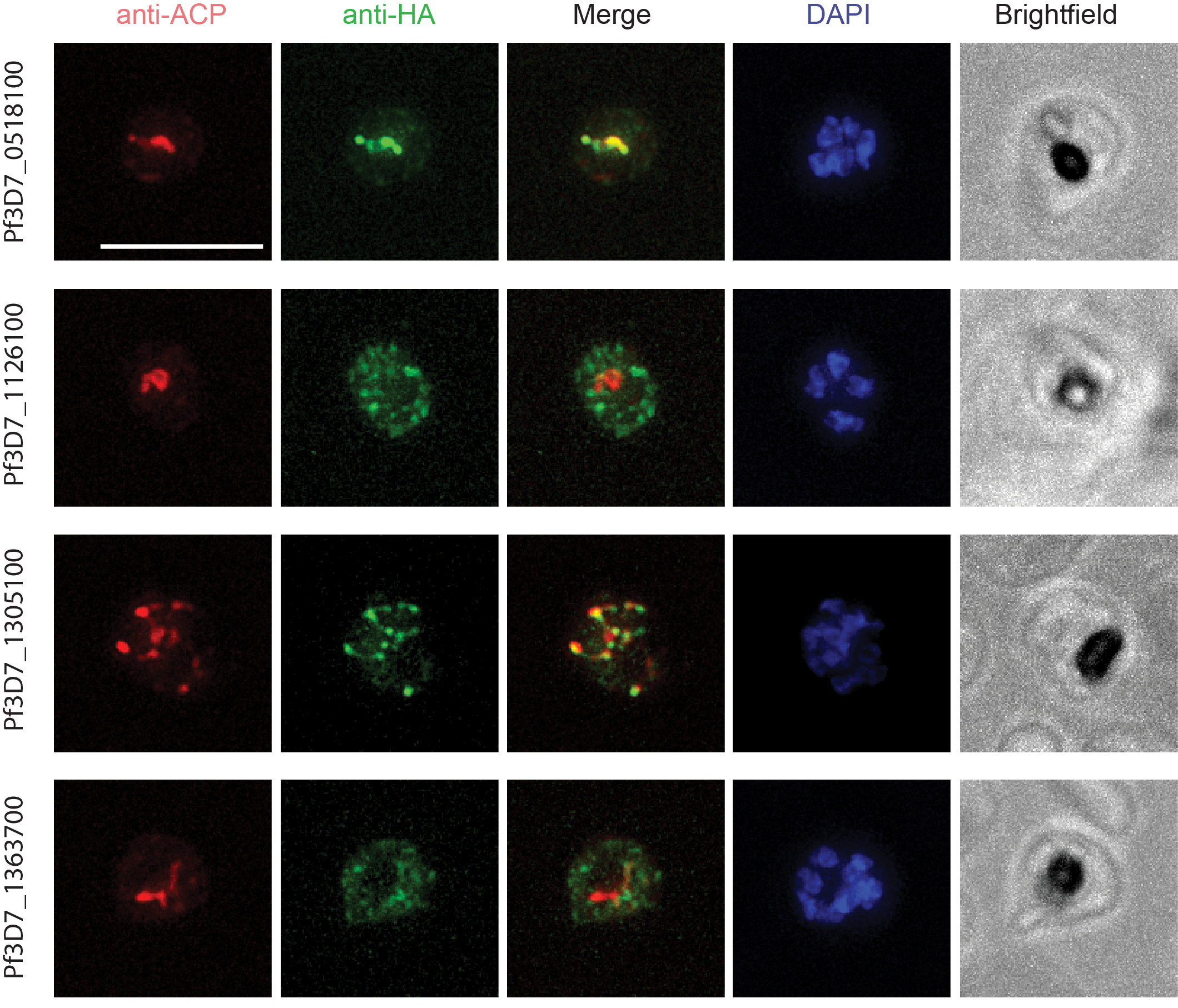

Supplement: S4 Fig — Representative immunofluorescence images of Pf3D7_0518100-TetR/DOZI, Pf3D7_1126100-TetR/DOZI (PfAtg7), Pf3D7_1305100-TetR/DOZI, and Pf3D7_1363700-TetR/DOZI parasites showing colocalization of the apicoplast luminal marker ACP with 3xHA tag. Of note, lack of colocalization of Pf3D7_1363700-3xHA with ACP may be due to low protein expression. Scale bar 5 μm. (TIF) [file pbio.3000136.s004.tif]

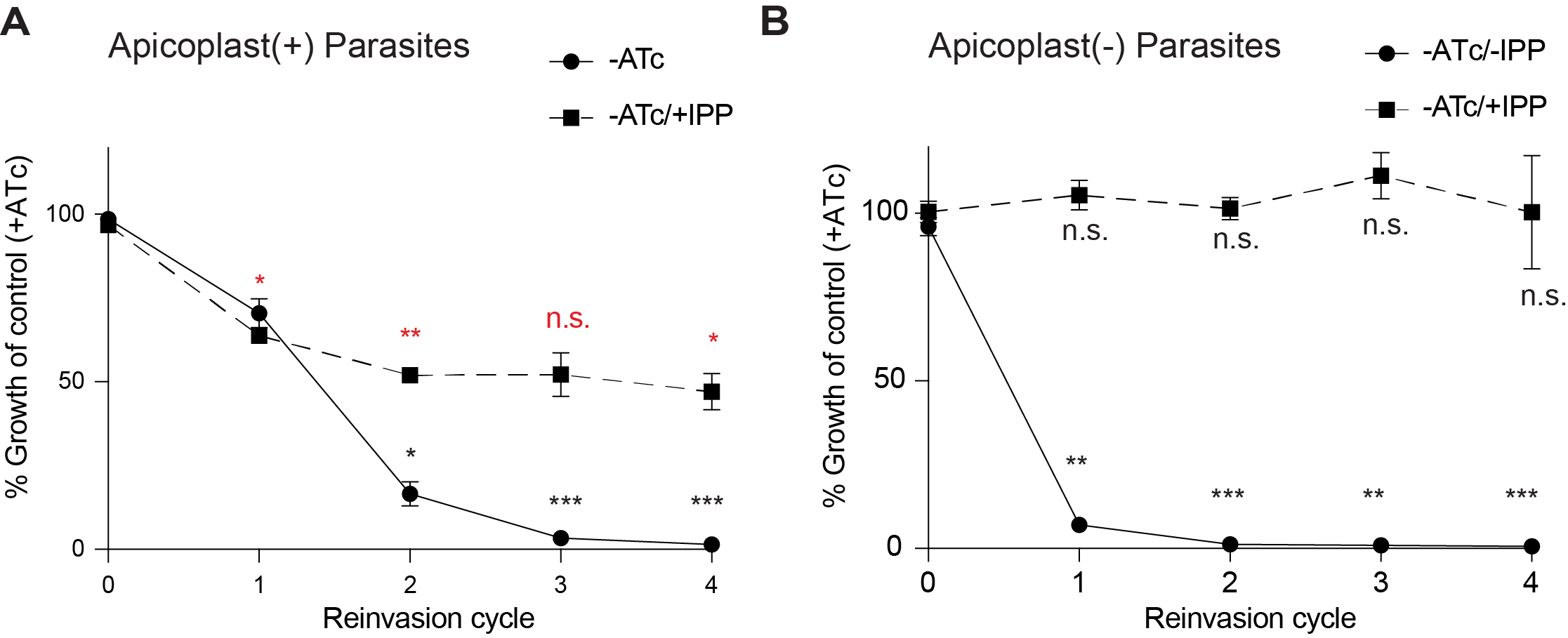

Supplement: S5 Fig — Growth time course of PfAtg7-TetR/DOZI parasites in absence of ATc with and without IPP. (A) PfAtg7 knockdown and IPP rescue precedes apicoplast loss in apicoplast(+) parasites. Data are shown as mean ± SD (n = 2). *P < 0.05, **P < 0.01, ***P < 0.001 compared to untreated control (−ATc black asterisks, −ATc/+IPP red asterisks), one-sample t test. Tabulated data are shown in S4 Data. (B) Apicoplast loss precedes PfAtg7 knockdown and IPP in apicoplast(−) parasites. Apicoplast(−) parasites were generated via actinonin/IPP treatment. Data are shown as mean ± SD (n = 2). **P < 0.01, ***P < 0.001 compared to untreated control (−ATc black asterisks), one-sample t test. Tabulated data are shown in S4 Data. (TIF) [file pbio.3000136.s005.tif]

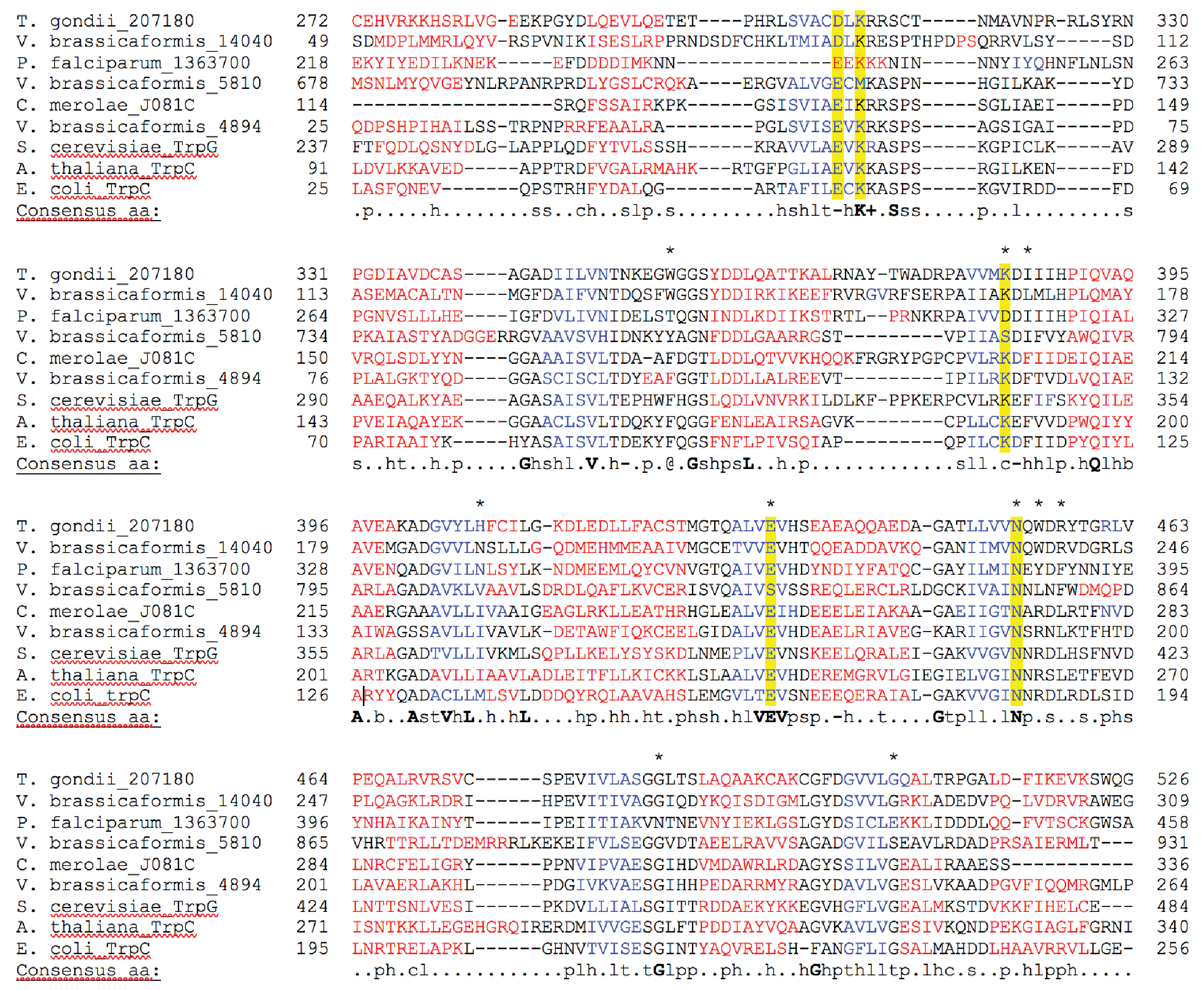

Supplement: S6 Fig — Residues involved in substrate binding and catalysis (based on the E. coli sequence) are marked with an asterisk and are highlighted in yellow, respectively. Blue and red residues represent predicted β-sheets and α-helices respectively. All other residues have no predicted secondary structure. Highly conserved residues are represented as bold uppercase letter in the consensus line. Other consensus symbols are as follows: b: bulky; c: charged; h: hydrophobic; p: polar; s: small; t: tiny; l: aliphatic; “+”: positive; “-”: negative; “@”: aromatic. (TIF) [file pbio.3000136.s006.tif]
