# Supplementary material for: A mutagenesis screen for essential plastid biogenesis genes in human malaria parasites
Source: PLoS Biol. 2019 Feb 6;17(2):e3000136. doi: 10.1371/journal.pbio.3000136 (PMC6380595; doi:10.1371/journal.pbio.3000136)
Supplement: S1 Table — (DOCX) [file pbio.3000136.s007.docx]

| Degron | Organism | Nucleotide sequence | Amino acid sequence | Reference |
| --- | --- | --- | --- | --- |
| ssrA | *E. coli* | gctgctaacgacgaaaa  ctacgctctggctgcttaa | AANDENYALAA | (33) |
| ssrA | *C. caldarium* | gcaaacaatattattgaaattagcaata  ttagaaaaccagctctagtagtc | AANNIIEISNIRKPALVV | (35) |
| X7 | N/A | aagctggcagctgcactggaa | KLAAALE | (34) |
